# Importance of proximity to resources, social support, transportation and neighborhood security for mobility and social participation in older adults: results from a scoping study

**DOI:** 10.1186/s12889-015-1824-0

**Published:** 2015-05-23

**Authors:** Mélanie Levasseur, Mélissa Généreux, Jean-François Bruneau, Alain Vanasse, Éric Chabot, Claude Beaulac, Marie-Michèle Bédard

**Affiliations:** 1 grid.86715.3d0000000090646198University of Sherbrooke, 2500 University Blvd., J1K 2R1 Sherbrooke, QC Canada; 2 grid.459289.b0000000102187524Research Centre on Aging, Health and Social Services Centre – University Institute of Geriatrics of Sherbrooke, 1036 Belvedere South, J1H 4C4 Sherbrooke, QC Canada; 3 Public Health Department, Health and Social Services Agency, 300 King East, Suite 300, J1J 1B1 Sherbrooke, QC Canada; 4 Montreal Polytechnique, Downtown Station, P.O. Box 6079, H3C 3A7 Montreal, QC Canada; 5 grid.411172.00000000100812808Research Centre, CHUS, 3001 12th Avenue North, J1H 5N4 Sherbrooke, QC Canada; 6 Ordre des urbanistes du Québec, H2Y 3V4, Montreal, QC Canada

**Keywords:** Neighborhood environment, Mobility, Social participation, Older adults, Quality of life, Scoping study

## Abstract

**Background:**

Since mobility and social participation are key determinants of health and quality of life, it is important to identify factors associated with them. Although several investigations have been conducted on the neighborhood environment, mobility and social participation, there is no clear integration of the results. This study aimed to provide a comprehensive understanding regarding how the neighborhood environment is associated with mobility and social participation in older adults.

**Methods:**

A rigorous methodological scoping study framework was used to search nine databases from different fields with fifty-one keywords. Data were exhaustively analyzed, organized and synthesized according to the International Classification of Functioning, Disability and Health (ICF) by two research assistants following PRISMA guidelines, and results were validated with knowledge users.

**Results:**

The majority of the 50 selected articles report results of cross-sectional studies (29; 58 %), mainly conducted in the US (24; 48 %) or Canada (15; 30 %). Studies mostly focused on neighborhood environment associations with mobility (39; 78 %), social participation (19; 38 %), and occasionally both (11; 22 %). Neighborhood attributes considered were mainly ‘*Pro ducts and technology*’ (43; 86 ) and ‘*Services*, *systems and policies*’ (37; 74 %), but also ‘*Natural and human*-*made changes*’ (27; 54 %) and ‘*Support and relationships*’ (21; 42 %). Mobility and social participation were both positively associated with Proximity to resources and recreational facilities, Social support, Having a car or driver’s license, Public transportation and Neighborhood security, and negatively associated with Poor user-friendliness of the walking environment and Neighborhood insecurity. Attributes of the neighborhood environment not covered by previous research on mobility and social participation mainly concerned ‘*Attitudes*’, and ‘*Services*, *systems and policies*’.

**Conclusion:**

Results from this comprehensive synthesis of empirical studies on associations of the neighborhood environment with mobility and social participation will ultimately support best practices, decisions and the development of innovative inclusive public health interventions including clear guidelines for the creation of age-supportive environments. To foster mobility and social participation, these interventions must consider Proximity to resources and to recreational facilities, Social support, Transportation, Neighborhood security and User-friendliness of the walking environment. Future studies should include both mobility and social participation, and investigate how they are associated with ‘*Attitudes*’, and ‘*Services*, *systems and policies*’ in older adults, including disadvantaged older adults.

## Background

Older adults make up a sizeable proportion of the population that will, between 2000 and 2050, double from about 11 to 22 %, including almost 400 million people worldwide aged 80 years or older [1]. Many people aged 65 and older suffer from chronic diseases such as arthritis and rheumatism (47.3 %), hypertension (42.8 %), heart disease (19.8 %) or diabetes (13.5 %), and almost half (42 %) have disabilities [2], which have significant consequences for individuals, communities, and social and health services. Chronic diseases and disabilities can be prevented or delayed by public health interventions (e.g., urban planning) as well as by clinical interventions (e.g., physician preventive practices) focusing on major modifiable health determinants. In comparison to the current population, future generations of older adults will likely have a better expectancy of years in good health [2] and, as a result, a larger proportion will have the potential for longer exposure to higher levels of mobility and social participation.

Social participation and mobility are major modifiable determinants of older adults’ health and key dimensions of successful aging [3]. On the one hand, mobility is broadly defined as “*the ability to move oneself* (*e.g*., *by walking*, *using assistive devices*, *or taking transportation*) *within community environments that expand from one*'*s home*, *to the neighbourhood*, *and to regions beyond*” [4]. It can be qualified in relation to life-space, from home to community. Mobility disability is common among older adults [5, 6]. For example, in Canada, more than 2.4 million people (10.5 %) [7] and approximately half of people aged 65 and older have restricted mobility [2, 4]. As a critical element of older adults’ health, diminished mobility has been associated with being physically inactive [8–11], obesity [8, 10, 12], physical disability [13–16], lower quality of life [13, 17, 18], premature mortality [19–21] and increased health care costs [22, 23]. Moreover, older adults participate more frequently in social activities if, especially when driving is not possible, they have access to private or public transportation. Community mobility using transportation, especially active or public transportation, is favorable to older adults’ health [24]. Sustainable modes of transportation simultaneously encourage physical activity and reduce local traffic-related pollution, both known to be associated with cardiovascular and other chronic diseases [25]. Access to public transportation for people living in rural areas may be limited, which can be a challenge [26]. Living in metropolitan, urban or rural areas can have an impact on many personal factors such as health and well-being, as well as on several environmental factors such as neighborhood socioeconomic status or access to services and transportation. To be closer to services, some older adults have moved from a rural to an urban area. In addition to individual factors such as health problems that affect muscle strength and balance, some environmental challenges such as constraints that involve physical loading and postural transitions (e.g., sloping terrain or stairs) can specifically influence mobility [27, 28].

On the other hand, social participation can be defined as “*a person*’*s involvement in social activities that provide social interactions within his*/*her community or society*” [29]. Specifically, social participation has been found to be a determinant of many favorable health and quality of life outcomes [30]. Identified as protecting against cognitive decline among community-dwelling older persons [31], social participation has been shown to be closely related to mobility in the community [32] and at home [33]. However, social participation declines as a result of the ‘normal’ aging process [34, 35] and, when limited, has been shown to be associated with mortality [36] and morbidity [37]. Greater disabilities and lack of neighborhood resources can restrict social participation [38] and decrease the likelihood of independent living [15]. In fact, disability, defined as any disturbance resulting from an impairment in the capacity to perform a physical or mental activity considered normal for a human being [39], has been found to be one of the most powerful determinants of social participation [40–50].

Interventions targeting environmental factors may have a greater impact on individual and population mobility and social participation than those targeting individual factors [51], including disability. The environment is defined by “*the physical and social characteristics in which people live*” [52] and, according to the International Classification of Functioning, Disability and Health (ICF) [53], includes five domains (chapters): 1) ‘*Products and technology*’, 2) ‘*Natural environment and human*-*made changes*’, 3) ‘*Support and relationships*’, 4) ‘*Attitudes*’, and 5) ‘*Services*, *systems and policies*’ (Appendix 1). Among the characteristics of the environment, neighborhood living conditions are important for health and well-being, especially for older adults. Compared to adults in the workforce, older adults are more place-bound [54, 55], i.e., spend more time each day in their neighborhood and stay longer in the same residential environment [2, 56]. Based on the definition of the physical environment of Davison and Lawson [57], neighborhood environment represents characteristics of the physical context including attributes of urban design (e.g., presence of sidewalks), traffic density and speed, distance to and design of venues for physical activity such as walking (e.g., parks and access to services), esthetics, crime and safety. Since mobility is also influenced by the social environment [4], i.e., ‘*Support and relationships*’, ‘*Attitude*’, ‘*Services*, *systems and policies*’, it is necessary to consider both physical and social neighborhood attributes and not only the built environment. Compared to younger adults, older people spend less time in structured employment activities and have more time to participate in other activities and be exposed to the neighborhood environment.

Since social participation and mobility can be enhanced [58], a clearer understanding of how environmental factors are associated with older adults is essential for informing and improving clinical [59] and public health [60] interventions such as age-friendly cities [61]. As illustrated by Lawton [51] and Glass and Balfour [56], two models widely used in public health, neighborhood *facilitators* (i.e., helpful factors, such as prostheses, resources and opportunities) can support personal capacities such as mobility, which can in turn enable greater social participation [51, 56]. In contrast, environmental *obstacles* (e.g., physical barriers, inaccessibility of services and amenities, social stress, and resource inadequacy) can challenge and exceed personal capacities, thereby limiting social participation. Support from the social environment [56, 62] and accessibility in the physical neighborhood environment [53, 56, 63–65] are seen as imperatives for helping individuals with disabilities living in the community [56, 66, 67].

Among neighborhood characteristics, living in close proximity to services [68, 69] has been shown to be important in performing activities to meet daily needs, including access to food shopping, health services, public transportation, banking and social clubs. Such proximity to services also contributes to initiating and maintaining social links with community members [69, 70]. Older adults living in resource affluent areas are less likely to have low levels of social functioning, independently of individual demographic (e.g. age) and socioeconomic (e.g. income) characteristics [71]. Individuals’ perceptions of the area as neighborly and having good facilities were also independently associated with a greater likelihood of social activities [71, 72] and well-being [73]. Walking distance, weather conditions, terrain characteristics, external physical loads, demands on attention, and traffic levels can all influence community mobility [13, 74–76] and social participation [77]. Finally, architectural (e.g., porches) and neighborhood design features can promote interaction among individuals in a neighborhood [78].

Despite the results of these studies and widespread acceptance of the importance of the neighborhood environment for mobility and social participation, a rigorous, integrative and comprehensive portrait is still lacking. Scoping studies are specifically designed to “… *identify gaps in the evidence base where no research has been conducted*” and to “… *summarise and disseminate research findings*” [79]. As for a systematic review, scoping methodology follows rigorous steps and a systematic process of study selection. This rigorous method considers both quantitative and qualitative research, and involves summarizing the results of studies to provide comprehensive evidence-based knowledge without specifically pooling the data or evaluating the quality of the studies. This scoping study thus aimed to provide a comprehensive understanding of how a wide range of physical and social neighborhood attributes is associated with or influences mobility and social participation in older adults. Such a synthesis of current knowledge represents an original contribution and may *ultimately* support decisions and the development of innovative interventions, clear guidelines and best practices regarding developing a neighborhood environment that enhances mobility and social participation in older adults.

## Methods

The methodological framework for scoping studies [79–82] was used to synthesize and disseminate current knowledge on the associations or influence of the neighborhood environment on mobility and social participation in aging [83]. The framework for the scoping study [79–82] includes collaboration between researchers and knowledge-users in the seven stages that were followed: i) identifying the research questions, ii) identifying relevant studies, iii) selecting the studies, iv) charting the data, v) collating, summarizing and reporting results, vi) consulting (throughout the project), and vii) dissemination of results.

### Identifying the research questions

Three questions were specifically addressed:

1. What are the social and physical attributes of the neighborhood environment which have been shown to be associated with or influence mobility and social participation in older adults?
2. How is the neighborhood environment associated with or how does it influence mobility and social participation in older adults?
3. Which attributes of the neighborhood environment have not been covered by previous research on mobility and social participation in older adults?

### Identifying relevant studies

The search involved nine databases (Medline, Cochrane Database of Systematic Reviews, CINAHL, Ageline, SocIndex, Psycinfo, Allied & Complementary Medicine Database (AMED), Academic Search Complete, *Francis*), fifty-one specific related keywords (Table 1) and targeted studies published in English and French between January 1980 and September 2013.

**Table 1:** Keywords chosen (n = 51)

Keywords [strategy: 1 AND 2 AND (3 OR 4)]‡	1. Built environment OR neighbourhood OR neighborhood OR environment* design* OR universal design* OR physical environment OR healthy environment OR living environment OR urban environment* OR suburban environment* OR rural environment* OR public transport* OR alternative transport* OR public transit OR paratransit OR bus OR buses OR urban design OR walkability OR walkable OR pedestrian OR social environment OR community design
	2. Elder* OR seniors OR old* adult* OR geriatric OR aged OR ageing OR aging OR older people
	3. Community participation OR social participation OR social involvement OR social engagement OR community involvement OR community engagement OR civic participation OR social isolation OR social integration OR social contact* OR social activity* OR social inclusion* OR social interaction* OR solitude OR loneliness OR lonely OR social exclusion*
	4. Mobility OR walking OR active transport*

‡To include all categories of keywords, the search strategy was more complex than presented here and is available upon request to the corresponding author

### Selecting the studies, charting the data, and collating, summarizing and reporting results

**Fig. 1:**
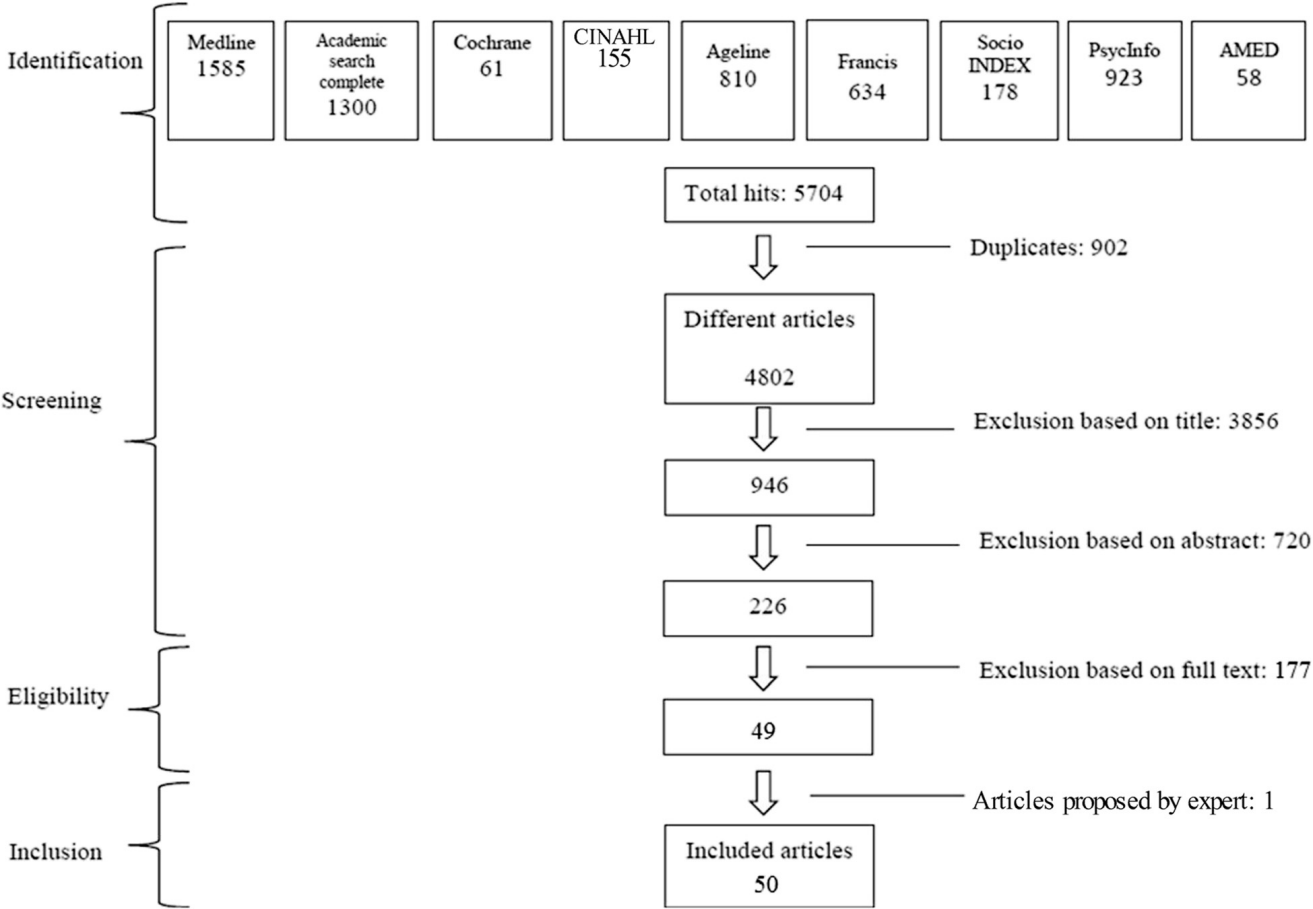
Flow chart

Two research assistants specifically trained and supervised by the principal researcher and information scientist, separately screened relevant articles by title and, when available, by abstract. To ensure transparency and reproducibility of the process [80], following PRISMA guidelines [84], all studies that comprehensively inform about the associations or influence of the neighborhood environment on mobility and social participation were retained and identified on a flow chart (Fig. 1). The selection of relevant literature was restricted, though not exclusively (retained if results specific to adults were also included), to papers on older adults. Extended search strategies included other studies found with a manual search of bibliographies and journals of interest (e.g., Health & Place, Annual Review of Public Health, and BMC Public Health). Relevant studies proposed by the team members and selected experts in the field of public health, rehabilitation and gerontology were also included (Fig. 1). Studies were excluded if they: 1) focused on narrow concepts (e.g., only on participation in a seniors’ centre or volunteering or home mobility, nursing home, gait, fear, migration, rehabilitation, physical functions, car settings, physical activity other than walking, daily activity, volunteering) or broader ones (e.g., exclusively on sociocultural, economic or policy attributes of the environment), 2) reported expert opinions or conference proceedings (often not providing sufficient information), or 3) concerned specific populations (e.g., people with diabetes or visual problems). The research assistants met regularly with the principal researcher and, at the beginning and in the middle of this process, with all team members to discuss and resolve any ambiguities concerning study selection, charting the data, or collating, summarizing and reporting results. An evolving data charting form [80] developed for this study and the definitions of all chapters of the environmental factors of the ICF (Appendix 1) [53] were used to classify the results independently extracted and categorized by the two research assistants and validated by the team. Content analysis procedures were followed where categories were grouped by meaning, synthesized, and then classified into coherent, consistent, relevant, clearly defined and productive themes [85]. This analysis also considered disadvantaged older adults, i.e., those with low income, minority status (e.g., race, ethnicity, gender, sexual orientation), limited education, frailty, or poor health (physical or mental). Such qualitative methods of analyzing documents ensure credibility and strength of the results [80]. Finally, a third team meeting was held to discuss the results with content experts and knowledge-users, identify implications and ensure clinical relevance of the results.

## Results

Of the 4802 papers retrieved through the electronic search, 49 met the inclusion criteria and one was added by the extended search strategies (Fig. 1). The year of publication of the papers ranged from 1997 to 2013 (Table 2). Half (number and percentage of papers: 25; 50 %) were published after 2009, with the most productive years being 2010, 2011, and 2012 (7, 8 and 8 respectively). About one third came from the field of gerontology (19; 38 %), another third from public health (17; 34 %) and approximately one fifth from rehabilitation (8; 16 %). Most papers exclusively concerned older adults (53; 86 %) and predominantly used the term neighborhood (27; 54 %) or environment (21; 42 %). The majority of the 50 selected articles reported results of cross-sectional studies (29; 58 %), mainly conducted in the US (24; 48 %) or Canada (15; 30 %), and a few were carried out with disadvantaged older adults, i.e., persons with disabilities (6; 12 %) or Low Neighborhood Socioconomic Status (2; 4 %; Table 2). Studies mostly focused on neighborhood environment associations with mobility (39; 78 %), social participation (19; 38 %), and occasionally both (11; 22 %). More than one third (18; 36 %) of the studies involved 150 participants or less, and about one fifth (11; 22 %) more than 1000. Most studies were carried out in urban settings (40; 80 %), and a few in rural (7; 14 %) or suburban (12; 24 %) areas (Table 2). Neighborhood measures were mainly subjective measures (34; 68 %), and sometimes objective (7; 14 %) or both (9; 18 %). Mobility (32; 82.1 %) and social participation (19; 100 %) were mostly self-reported measures, the former most commonly operationalized by walking (38; 94.4 %), but also sometimes focusing on driving (10; 59.0 %) or active and alternative transportation (13; 33.3 %).

**Table 2:** Characteristics of the articles on neighborhood environment, mobility and social participation in older adults

Reference number	Country	Setting	Design	Population (sample size; age)	Objective
[25]	USA	Suburban	Cross-sectional	1970; 65+ (65–85+)	To assess the relationship between urban form and walking, driving, physical activity, food access, and weight status in a large sample of older adults responding to a travel survey.
[27]	Canada USA	Urban	Cross-sectional	36; 70	To examine environmental challenges encountered by older adults without mobility impairments while walking in the community.
[71]	Europe	Urban, rural	Cross-sectional	761; 65–85+	To investigate associations between socioeconomic characteristics of the area, perceived neighborhood and indicators of social and physical functioning.
[72]	USA	Urban	Longitudinal	1821; 45–92	To examine adult trajectories of mobility disability over the 15-year study period (1986–2001).
[86]	Europe	Urban rural suburban	Cross-sectional	48,879; <65	To 1) investigate the relationship between area of residence and walking and cycling for transportation and recreation in Flemish older adults, and 2) study the relationship between several physical environmental factors and walking, and cycling and possible moderating effects of area of residence, age and gender.
[87]	Europe	Urban rural suburban	Qualitative	21; 82–90	To describe how very old people experience occupational performance outside the home.
[88]	Canada	Urban	Cross-sectional	296 women, 258 men; 75 (67–84)	To examine (1) the association between neighborhood environment, specifically perceived proximity to neighborhood resources, and social participation above and beyond disability; and (2) the moderating effect of this neighborhood variable on the association between disability and social participation in older women and men living in an urban area.
[89]	USA	Urban	Cross-sectional	1225; 45–92	To examine the role of certain characteristics in the urban built environment as they interact with underlying impairments and activity limitations either to promote or hinder the participation of adults in society.
[90]	Asia	Urban	Cross-sectional	484; 65–74	To examine 1) the associations of objectively-measured prevalence and diversity of nine destination categories with overall and within-neighborhood walking for transport in Chinese elders residing in Hong Kong, an ultra-dense metropolis, and 2) the moderating effects of neighborhood safety and pedestrian infrastructure aspects on the above associations.
[91]	Europe	Urban	Cross-sectional	4899; 12+	To investigate whether physical activity is an underlying mechanism in the relationship between the amount of green space in people’s direct living environment and self-perceived health.
[92]	Canada USA	Urban	Cross-sectional	54; 70+	To examine the relationship between characteristics of the physical environment and mobility disability in community-living older persons.
[93]	Canada	Urban	Qualitative	486; 20–75+	To assess group perceptions regarding ease of movement in a town centre and accessibility to premises.
[94]	Canada	Urban	Cross-sectional	2614; 45+	To examine the association between neighborhood active living potential and walking among middle aged and older adults.
[95]	USA	Urban	Cross-sectional	582; 64–94	To explore the influence of neighborhood-level characteristics on elderly physical activity.
[96]	USA	Urban	Cross-sectional	546; 65+	To examine the relationship between objectively measured characteristics of the local neighborhood and walking activity among community-dwelling older adults in Portland, Oregon.
[97]	USA	Urban	Cross-sectional	1195; 45–92	To examine the effect of block-level built environment characteristics on mobility disability among adults aged 45 and over who vary in their level of lower extremity physical impairment.
[98]	Canada	Urban	Cross-sectional	60; 65+	To examine the associations between walking behavior and the perceived environment and personal factors among older adults living in a downtown neighborhood of a midsized Prairie city.
[99]	Canada USA	Urban	Qualitative	66; 65+	To identify neighborhood social and physical environmental aspects that influence older adults’ physical activity.
[101]	USA	Urban suburban	Cross-sectional	251; 65+	To explore the ability of neighborhood design to preserve accessibility for the elderly by enabling a shift from driving to transit and walking, while controlling for neighborhood preferences and attitudes towards transportation.
[102]	Canada	Urban suburban	Qualitative	75; 65	To understand older people’s neighborhood walking experiences with an emphasis on daily life.
[105]	USA	Not reported	Qualitative	60; 55+	To answer the research question: How does neighborhood design encourage or inhibit active aging according to older adults?
[106]	Canada	Not reported	Cross-sectional	200; 65+	To examine the effect of the environment on participation while controlling for the individual’s personal factors
[107]	USA	Urban	Qualitative	7; 55+	To identify the strategies used to create and maintain social participation for older adults living alone in the community, and explore older adults’ own perceptions of their experience of social participation.
[108]	USA	Urban	Longitudinal	217; 70+	To examine the longitudinal relationship between perceived neighborhood climate and walking behavior, over a 12-month period
[109]	Asia	Urban	Cross-sectional	484; 65–74	To examine associations of perceived neighborhood environmental attributes believed to influence walking with overall and within-neighborhood recreational walking in a sample of Chinese elders residing in an ultra-dense metropolis with a developed public transport system (Hong Kong).
[110]	Europe	Rural suburban	Qualitative	42; 65–79	To obtain a qualitative assessment of the opinions of the elderly living in rural areas regarding their leisure and recreational habits.
[111]	Brazil	Not reported	Cross-sectional	1652; 60+	To evaluate the association between safety from crime and physical activity among older adults
[112]	Europe	Urban	Longitudinal	261; 75–81	To identify the effect of environmental facilitators for outdoor walking on development of walking difficulty in community-dwelling older people.
[113]	Europe	Not reported	Qualitative	957; 81.7	To describe older people’s motive for and experiences of mobility and occupational participation outside the home.
[114]	USA	Urban	Qualitative	21; 60+	To identify the salient factors of the neighborhood environment that encourage or discourage walking in older, urban African Americans.
[115]	Canada	Urban suburban	Longitudinal	521; 67–84	To examine whether or not closer proximity to local services and amenities was associated with maintenance of more frequent walking over time among urban-dwelling seniors over and above individual-level characteristics.
[116]	Asia	Urban rural suburban	Cross-sectional	1921; 65–74	To examine the association between perceived neighborhood environment and walking for specific purposes among Japanese elderly adults.
[117]	USA	Not reported	Longitudinal	438; 65+	To examine participation in 2 areas: (1) social and home participation, which is related to self-care and domestic functioning, financial functioning, social relationships, and communication; and (2) community participation, which reflects participation related to a person’s mobility, functioning in work, and other ADLs.
[118]	USA	Urban	Cross-sectional	91; 68.7 (64–91)	To explore the possibility that older adult’s exposure to green common spaces is related to an increased sense of local community because of enhanced levels of social integration.
[119]	USA	Urban	Longitudinal	303; 65+	To examine change in neighborhood walking activity over a 12-month period in a community-based sample.
[120]	USA	Urban	Cross-sectional	577; 74	To examine the relation between built environment factors and walking activity at both the neighbourhood level and the resident level, in an older adult population.
[121]	Europe	Urban rural	Cross-sectional	90 neighborhoods; 45–73	To analyze the impact of the neighborhood on individual social capital.
[122]	Canada	Urban rural	Qualitative	22; 76 (60–90)	To examine environmental factors influencing the walking choices of elderly people.
[123]	Canada	Suburban	Qualitative	22; 62–89	To 1) illustrate participants’ typical day in order to identify changes since 1999, that is, the strategies of ‘*déprise*’ (abandonment) and their impact on daily mobility; 2) reveal the experiences and meanings of “home” in light of changes in daily mobility during a six-year period, and with regards to elders’ representations of the city and of aging; 3) shed light on individual reasons behind territorial mobility adaptation strategies and describe the relationship of elderly to the broader urban environment.
[124]	USA	Urban	Cross-sectional	4317; 65+	To examine individual differences in walking behavior among community-dwelling older adults in relation to two features of the neighborhood environment—social cohesion and exchange, and neighborhood disorder.
[125]	USA	Urban	Cross-sectional	105; 65+	To examine the degree of association between perceived and objective characteristics of the neighborhood environment and the relation of each type of measurement to neighborhood walking in older adults.
[126]	USA	Urban suburban	Cross-sectional	372; 70+	To explore the relationship between pedestrian-friendly urban form as reflected in new urbanism design guidelines, and neighborhood service use, walking, driving, quality of life, and neighborhood satisfaction among older women.
[127]	Canada	Urban	Cross-sectional	282; 58+	To investigate the relationship between perceptions of neighbourhood user-friendliness and social participation.
[128]	Canada	Urban		520; 67–84	To examine the associations between proximity to selected locations considered to be conducive to social participation, and social participation itself, in urban-dwelling seniors.
[129]	USA	Urban	Qualitative	37; 55+	To determine perceptions of environmental supports for and barriers to walking and biking behavior in older adults and to evaluate whether perceptions differed by defined neighborhood walkability.
[130]	Canada	Not reported	Cross-sectional	350; 65+	To compare participation of older adults according to the level of urbanization of their home environment, and to explore sociodemographic factors associated with participation in relation to the urbanization level of their environment.
[131]	Europe	Urban	Qualitative	24; 55–87 (mean 75)	To explore the behavior and representations of seniors concerning doing physical activities to identify obstacles to going out and walking, their need to overcome these obstacles over the long term, and communication channels to disseminate information about a walking route (*translation*).
[132]	Europe	Urban suburban	Qualitative	57; 65+	To identify the perceived environmental influences on Flemish older adults’ walking for transportation.
[133]	USA	Not reported	Cross-sectional	436; 65+ (mean 70.4)	To explore the association of particular features of neighborhood environments with disability among older adults with existing functional limitations.
[134]	USA	Urban suburban	Qualitative	38; 62–85	To understand how older adults perceive and navigate their neighborhoods, the study looked at the implications of activity in their neighborhoods for their health to identify the types of resources that people use in their residential settings to maintain or improve their overall well-being.

Neighborhood attributes considered were mainly ‘*Products and technology*’ (43; 86 %; Table 3) and ‘*Services*, *systems and policies*’ (37; 74 %), but also ‘*Natural environment and human*-*made changes to environment*’ (27; 54 %) and ‘*Support and relationships*’ (21; 42 %). Among the 103 attributes studied, the majority were positively (see + in Table 3; 62; 60.2 %) associated with mobility or social participation. Associations of mobility or social participation with neighborhood attributes were primarily positive (209; 54 %; Table 3), but some were negative (86; 22.2 %) or non-existent (92; 23.8 %). Twenty-two divergent associations were found among the same studies, contrasting specific contexts such as people with disabilities versus without, walking versus driving. Attributes of the neighborhood environment not covered by previous research on mobility or social participation mainly concerned ‘*Attitudes*’, and ‘*Services*, *systems and policies*’ (Appendix 2).

**Table 3:** Synthesis of literature review of environmental factors positively (+), negatively (−) or not (0) associated with mobility and social participation in older adults

Environment	Mobility	Social participation
**Chapter 1: Product** ^*^ **and technology**		
*e120: Products and technology for personal indoor and outdoor mobility and transportation*		
Mobility assistive device	+[87], −[123]	+[87], 0[117]
*e125: Products for communication*		
Communication technology	+[113]	+[113], 0[117]
*e140: Products and technology for culture, recreation and sport*		
Absence of parks and walking areas	−[111]	−[133]
Community gardens	+[99]	+[99]
Space for socialisation	+[86], +[87], +[99], +[102]	+[87]
*e150: Design, construction and building products and technology of buildings for public use*		
Absence of high ramps	0[132]	
Adequate handicap parking	+[123], +[133]	+[123]
Buildings difficult to access	−^†^[93]	
Escalators, curbs and uneven surfaces	0^‡^/–^§^[92]	
Parking	+[93], +[99]	
Public facilities	0[90], +[114]	
Seating	+[86], +[87], +[93], +[99], +[102], +[105], 0/+^**^[109], +[122], +/0[132]	+[87], +[105], 0[133]
Toilet facilities adequate for persons with disabilities	+[93]	
Universally accessible public spaces	+[99],+[123]	+[123]
Washrooms	+[99], +[122], 0[132]	
Water fountains	+[99], +[122]	
*e155: Design, construction and building products and technology of buildings for private use*		
Easy access of residential entrance	0[109]	
Home architectural mobility barriers		−^††^[117]
*e160: Products and technology of land development*		
Aesthetics^‡‡^	+[86], 0[98], 0[109], +[114], +[116], +[122], +[129], +[132]	
Bad condition of sidewalks	+[114]	
Bridge/overpasses connecting to services	+[109]	
Crossing	+[86], +[132]	
Dispersion of resources	−[110], −[129]	−[110]
Fence separating sidewalks from traffic	0[109]	
Few streets	−[96]	
Good condition of streets/path	+[87], +[90], 0[97], +[99]	
Good quality of facilities		+[71]
Good user-friendliness of the walking environment	+[72], 0[94], +[113], +[135]	+[113], +[127], 0[128]
Indoor shopping areas	+[114]	
Mixed services and good pedestrian access	+[126]	
No curbs with curb cuts		0[133]
Uninviting neighborhood surroundings	-[114]	
Poor user-friendliness of the walking environment	0^‡**^/−^§††^[27], −[87], 0[90], −[102], −[105], 0[109], −[113], −[115], −[122], −[131]	−[99], −[105], −/+^††^[107], −[118]
Poorly maintained or missing sidewalks, crosswalks, bike paths or lanes	−[129]	
Garbage	−[111]	
Proximity to recreational/exercise facilities	0/+^1^[90], +[95], +[99], +[105], +[109], +[112], +[113], +[114], +[116], 0[119], +[120], +[122], 0[125], +[132]	+[105], +[113]
Relocation of community services and shops	−[102], −[113]	−[102],−[113]
Resources proximity	+[86], 0/+^1^[90], +^††^[93], 0^††‡‡‡^/+[94], +[96], 0[98], +[99], +^§***†^/−^†††^[101],+[102], +[105], 0/+[115], +[116], +[120], +[123], +[125], +[126],+[129], +[134]	+[88], +[105], +[107], +[110], +[123], +[127], +[128]
Rural > urban	+^***^[101]	
Safe stairs	+[99]	
Sidewalks	+[86], 0[96],+[99], +[102], +[105], 0[111], +[114], 0[116], 0[125]	+[105]
Streets connectivity	+[86], 0[96], 0[98], 0[109]	
Streets in poor condition		−^§††^/0[89], −^§††^/0^‡**^[97]
Streets with traffic lights and busy streets	0^‡**^/−^††^[92]	
Traffic and road hazards	0[109]	
Traffic lights located at inconvenient spots	−[122]	
Unfamiliar places	0^‡**^/−^††§^[92]	
Urban > rural	+^†§^[101], 0^‡‡‡^/+^§§§^[132]	+^†‡‡^[101]
Urban > semi-urban > rural		+[110], 0[130]
Walking/cycling facilities	0[98],+[109], 0[111], −^3^/+[114], +[116], 0[125], +[129]	
*e165: Assets*		
Packages carried	−[27]	
**Chapter 2: Natural environment and human-made changes to environment**		
*e210: Physical geography*		
Topography physically demanding	0[90], 0[111], −[113]	−[113]
*e215: Population*		
Crowded places with high traffic density	0^‡**^/−^††§^[92]	
Living in prosperous areas		+[71]
Low level of traffic	+[101]	
Low Neighborhood Socioeconomic Status	+[95], +[96]	
Neighborhood		+[121]
Population density	0[72], 0[109], 0[116], +[120]	
Seniors density	0[72], +[95]	
Traffic	+[96],−[105], 0[109], 0[111],−^***^[113], −[132]	+^††§****^[89],−[105],0[113]
White people density	+[95]	
*e220: Flora and fauna*		
Animals	−/+^2^[114]	
Stray animals	−[90]	
Lack of greenery	−[131]	
Nature and green space	+[86], 0[91], +[93], +[102], 0[111], +[114], +[129], 0[132]	+[118]
*e225: Climate*		
Poor weather conditions	0^**‡^/−^§††^[92], −[102], 0[111], −[113], −[114], −[122]	−[113]
*e240: Light*		
Inadequate street lighting	−[92], −[111], −[114]	
Street lighting	−[27], +[86], +[90],+[99], +[132]	
*e245: Time-related changes*		
Night time	−[113]	−[113]
*e250: Sound*		
Absence of noise	+[132]	
*e260: Air quality*		
Fresh air	+[114]	
Open sewers	0[111]	
Smoke pollution	0[111]	
**Chapter 3: Support and relationships**		
*e310: Immediate family*		
Support from family	+[87], +[123]	+[87], 0[106],+[123]
*e320: Friends*		
Support from friends	+[123]	0[106],+[123]
*e325: Acquaintances, peers, colleagues, neighbors and community members*		
Children living in the neighborhood	−[115]	0[128]
Lack of social support	−[113]	−[113]
People	+[86], +[102], 0[109], +[114], +[116], +[129]	+[99], +[107]
Social cohesion	+[95], +[119], 0[124]	
Social support/network	+[99], 0[111], 0[115],+[134]	+[71], +[107], +[117], +[128]
Walking partner	+[102]	
*e345: Stranger*		
Crowdedness	0[109], −[114]	
*e350: Domesticated animals*		
Not having or not walking a dog	−[111]	
**Chapter 4: Attitudes**		
*e445: Individual attitudes of strangers*		
Drivers’ respect for pedestrians on crossings	0[111]	
Negative attitude of people	−[87]	−[87]
Disrespectful attitude of bus drivers	−[113]	−[113]
*e460: Societal attitudes*		
Poor walking culture & sidewalk etiquette	−[102]	
**Chapter 5: Services, systems and policies**		
*e515: Architecture and construction services, systems and policies*		
Architectural features that facilitate social contacts	+[108]	
*e525: Housing services, systems and policies*		
Retirement home/housing facilities	+[123]	+[123]
*e540: Transportation services, systems and policies*		
Car or driver’s license	+[87], +[99], +^***^/−^†§^[101], +[102], +[113], 0[115]	+[71], +[87], +[113], +[127], 0[128], +[130],+[134]
Inadequate public transportation	−[110], −[113]	−[113], −[110]
No or only one car for the dwelling	+^†††^[101], +^††††^/0[116], +^†§^[135]	
Protection and comfort at bus stops	+[93]	
Public transport	+[86], 0[90], +^††§^[93], 0[96], +[99], + [101], +[102], +[105], +[113], 0[115], 0[116],+[122], +[132], +[134]	0[89],+[105], +[107], +[113], 0[128], +[133]
Transportation facilitators		+[117]
*e545: Civil protection services, systems and policies*		
Graffiti	-[99], -[125]	
Neighborhood security	+[86], +[90], +^§††^[93], 0[94], 0[95], 0[98] + [99], +[101], 0[111], +[114], +[119], +[120], +[122]	0[71], +^§††^[89]
Neighborhood insecurity	0[90], 0[95], −[96], 0[109], −[113], −[114], +^††††^[116], −[124], −[132]	0[71], −[89], −[113], 0[133],
Traffic-related safety	+[86],+[99], 0^††††^[116], +[122]	+[89]
Traffic-related insecurity	−[129]	
*e555: Associations and organizational services, systems and policies*		
Community-based programs	+[99]	
*e560: Media services, systems and policies*		
Virtual and media-related mobility	−[113]	−[113]
*e580: Health services, systems and policies*		
Promotion of sports and/or walking events	0[111]	

*Article or substance that is manufactured or refined for sale. This definition and the chapters are based on the International Classification of Functioning, Disability and Health (ICF) [53]

^†^For walking

^‡^For persons without disability

^§^For persons with disabilities

^**^For seniors 75 years old and older

^††^Particularly in the period shortly after discharge from an acute care or inpatient rehabilitation hospital

^‡‡^Concerned with beauty or the appreciation of beauty

^§§^No signs of crime/disorder

^***^For driving

^†††^For public transportation

^‡‡‡^Weekly recreational walking/cycling

^§§§^Walk daily for transportation

^****^Authors explained that heavy traffic is associated with greater interpersonal interactions (perhaps because these areas also tend to have more public transit stops (not captured by our measure of proximity to public transit lines) or cafes and restaurants that facilitate interactions

^††††^For men walking for active transportation

^1^Depending on resources, their proportion or their diversity

^2^Depending if they enjoy them or are afraid of them

^3^If dangerous for crime

Selected studies considering ‘Products and technology’ (Table 3) mainly focused on ‘*Products and technology of land development*’ (43; 86 %) and ‘*Design*, *construction and building products and technology of buildings for public use*’ (14; 28 %). From these studies, mobility and social participation were both principally positively associated with Seating, Good user-friendliness of the walking environment and Proximity to resources and to recreational facilities, and negatively associated with Poor user-friendliness of the walking environment. Space for socialization, Esthetics, Good condition of streets/paths, Sidewalks and walking/cycling facilities were also positively associated with mobility, while Streets in poor condition was negatively associated with social participation (Table 3).

Among ‘*Natural and human*-*made environment*’, studies considered principally ‘*Population*’ (15; 30 %) and ‘*Flora and fauna*’ (11; 22 %). Mobility was mainly positively associated with Nature and green space, and Street lighting, and negatively with Traffic and Poor weather conditions (Table 3). Studies on ‘Support and relationships’ focused on ‘*Acquaintances*, *peers*, *colleagues*, *neighbors and community members*’ (18; 36 %) and found that People and Social support/network were both positively associated with mobility and social participation. As very few of them concerned ‘*Attitudes*’, no association was confirmed by more than one study (Table 3). Finally, studies on ‘*Services*, *systems and policies*’ mainly considered ‘*Transportation services*, *systems and policies*’ (25; 50 %) and ‘*Civil protection services*, *systems and policies*’ (24; 48 %). Mobility and social participation were both mainly positively associated with Having a car or driver’s license, Public transportation and Neighborhood security, and negatively with Neighborhood insecurity (Table 3). No or only one car for the dwelling and Traffic-related safety were associated, respectively, positively and negatively with mobility.

## Discussion

This study provided a comprehensive understanding of neighborhood environment associations with mobility, i.e. the ability to move oneself within community environments [4], and social participation, i.e. a person’s involvement in social activities that provide social interactions within his/her community or society’ [29], in older adults. Mobility and social participation were both mainly positively associated with Proximity to resources and to recreational facilities, Social support, Car or driver’s license, Public transportation and Neighborhood security, and negatively with Poor user-friendliness of the walking environment and Neighborhood insecurity. For example, living in close proximity to services [68] was shown to be important in performing activities to meet daily needs, including access to food shopping, health services, public transportation, banking and social clubs, and initiating and maintaining social links with community members [70]. Older adults living in resource affluent areas are less likely to have low levels of social functioning, independently of individual demographic and socioeconomic characteristics [71]. Moreover, having sufficient and convenient local business stores in the neighborhood allows older adults to remain active, which is beneficial for their health and may lead to longer independent living. The absence or disappearance of local businesses making it impossible for older adults to walk to these resources is a concern [86], especially when they prefer or are restricted to the immediate neighborhood [87]. Such results highlight the importance of urban planning interventions for neighborhood revitalization and for survival of proximity resources, limiting the creation of large supermarkets far from people’s homes [88]. Such an absence is worrying since it is known that more proximate characteristics in one’s immediate environment are more salient than characteristics in the wider neighborhood area [89].

Although associations of mobility and social participation with resource proximity were usually positive, few non-existent associations with mobility were found, illustrating the complexity of this type of study. One study found that the effects of neighborhood attributes on within-neighborhood recreational walking were stronger in less educated participants [90]. In another study, mobility was associated with greater diversity in recreational destinations only in neighborhoods with no signs of crime/disorder or stray animals [90]. Food and grocery stores were also associated with mobility, at least in the absence of path obstructions or sloping streets. In fact, the availability of resources may promote within-neighborhood walking for transportation, while recreational facilities and public transit points may facilitate overall walking [90]. However, destination-rich neighborhoods also need to provide a safe and physically unchallenging walking environment. Complexity is also highlighted by the fact that in green space living environments, facilities such as shops are further away and people use a car more often to reach resources [91]. For instance, interaction between neighborhood effects and individual characteristics, as described in the Glass and Balfour model, may be observed.

Moreover, this study highlighted the fact that few studies considered the context of persons with disabilities, which warrants further special attention. Such a context was particular and different. For example, contrary to people without disabilities, the mobility of persons with disabilities was negatively associated with neighborhoods having escalators, curbs, uneven surfaces, streets with traffic lights and busy, crowded places with high traffic density (people or objects), as well as poor weather conditions (snow and ice; cold and rainy) and unfamiliar places [92]. One study found that mobility of disadvantaged older adults was positively associated with it being safe to walk, public transportation and proximity of resources [93], while another did not support this latter association [94]. Low neighborhood socioeconomic status was positively associated with mobility [95, 96]. Social participation of persons with disabilities was negatively associated with neighborhoods with streets in poor condition [97], but positively with traffic and residential security [89]. Finally, these conflicting results might suggest that among older adults with disabilities, mobility was more related to personal and intrinsic physical capacities than to the perceived environment [98]. Future research should focus on the context of persons with disabilities.

As it is critical to consider not just how older adults use resources but also how they get to them [99], more neighborhood studies on both mobility and social participation are needed. Even if the best resources are available, older adults, especially those with varying mobility challenges, will not use them if they cannot get to them easily and safely. First, public transportation including adequate public transit or other shared options is critical [99], especially for older adults who cannot walk long distances or have stopped driving. Social exclusion of older adults is reinforced by an inadequate public transit system or one that cannot adequately serve the entire municipality [100]. Although it is not a preferred mode for older adults having a car and a driver’s license [101], there is a need to develop a more efficient public transit system since the location of resources can only change slowly. Second, seeing other people or social support is important. More alternative transportation solutions and personalized accompaniment to activities might also foster mobility and social participation. Since older adults might be more likely to be mobile or participate when activities are meaningful to them [99], the impact of seeing other people walking or doing social activities should not be underestimated. Such surroundings help to prevent victimization and provide assistance in case of a health emergency or fall [102]. Moreover, integrating older adults into their community can provide them with emotional support, motivation, information, social interaction, friendship, sense of belonging, etc.

### Strengths and limitations

Based on an international classification considering a wide range of environmental attributes, this study used a rigorous methodological framework for scoping studies [79–82], including a systematic and comprehensive retrieval of studies on the neighborhood environment, mobility and social participation from numerous multidisciplinary databases. In addition, results from quantitative studies were completed and extended by results from qualitative studies [103], which help to understand how the neighborhood environment influences mobility and social participation. Enriched by the close collaboration of knowledge-users from different fields (public health, urban planning, transportation planning, rehabilitation and gerontology) in a variety of institutions (academic, health and social services agencies, public transit authorities and municipalities), the results provide an accurate and up-to-date synthesis of the literature on how the neighborhood environment is associated with or influences mobility and social participation in older adults. Moreover, attributes not covered by previous research on the influence of the neighborhood environment on mobility and social participation were identified to inform future interdisciplinary research. However, as in other scoping studies [79], the current study does not systematically combine empirical results of previous studies or provide a detailed appraisal of the quality of the evidence. Furthermore, although the impact of not using textbooks should be minimal since they are generally not a primary source of empirical results, information available in them may have been missed. Although carefully reviewed and identified by two research assistants, retrieval of studies on the neighborhood environment, mobility and social participation was challenging as there are numerous associated key words and some of them (e.g., walk) generated many irrelevant results. Finally, as definitions and measures of neighborhood environment, mobility and social participation differ greatly across studies, results should be interpreted with caution although the synthesis involved many specifications.

## Conclusion

Results from this comprehensive synthesis of empirical studies on the association of the neighborhood environment with mobility and social participation may ultimately support best practices, decisions and the development of innovative inclusive public health interventions including clear guidelines for the creation of age-supportive environments. To foster mobility and social participation, these interventions must consider Proximity to resources and to recreational facilities, Social support, Transportation, Neighborhood security and User-friendliness of the walking environment. These results will ultimately help to promote community-driven development [104] or active living in older adults, which are among the main goals of public health specialists. For example, decision-makers in the municipality can use results from this scoping study to support projects or make decisions about financial investments in urban planning and public safety (modifications to the neighborhood environment that encourage mobility and social participation). This information will also be useful for making policy recommendations related to land use planning and transportation, to assist in senior-friendly developments, redevelopments, revitalization plans and neighborhood improvements, and to design effective senior health interventions with an emphasis on neighborhood design influences and their location [105].

Future studies should examine mobility and social participation simultaneously, and investigate how they are associated with ‘*Attitudes*’, and ‘*Services*, *systems and policies*’ in older adults, including in disadvantaged older adults. This scoping study represents the first stage of a research program to: 1) identify key age- and gender-specific neighborhood environment determinants of mobility and social participation, controlling for individual factors such as tobacco use, body composition (obesity, nutrition) and energy expenditure (physical exercise); 2) develop health-related analytical geomatic tools (interactive atlas) that monitor these relevant neighborhood environmental features from extended continuous recordings; and 3) develop efficient knowledge transfer protocols for clinicians and decision-makers in the form of better clinical toolkits (scales or portable devices) for assessing the impact of intervention strategies on mobility and social participation. Finally, future studies on mobility and social participation need to use innovative ways to collect data. In addition to Photovoice [99] and Walk-along interviews to and from a destination (e.g. a shop) located within a 15-min walk from the participant’s home [86] used previously, increasingly a geographic information system should be used [88]. These studies will eventually lead to the development of specific intervention strategies, including more comprehensive legislation and policies that can prevent mobility and social participation inequalities by optimizing neighborhood environment issues to improve health and quality of life in the population in general and especially in the older population.

## Appendix 1

Definitions of environmental factors according to the International Classification of Functioning, Disability and Health (ICF) [53].

Chapter 1: Products and technology: This chapter is about the natural or human-made products or systems of products, equipment and technology in an individual’s immediate environment that are gathered, created, produced or manufactured. The ISO 9999 classification of technical aids defines these as “any product, instrument, equipment or technical system used by a disabled person, especially produced or generally available, preventing, compensating, monitoring, relieving or neutralizing” disability. It is recognized that any product or technology can be assistive. (See ISO 9999: Technical aids for disabled persons—Classification (second version); ISO/TC 173/SC 2; ISO/DIS 9999 (rev.).) For the purposes of this classification of environmental factors, however, assistive products and technology are defined more narrowly as any product, instrument, equipment or technology adapted or specially designed for improving the functioning of a disabled person.

Chapter 2: Natural environment and human-made changes to environment: This chapter is about animate and inanimate elements of the natural or physical environment, and components of that environment that have been modified by people, as well as characteristics of human populations within that environment.

Chapter 3: Support and relationships: This chapter is about people or animals that provide practical physical or emotional support, nurturing, protection, assistance and relationships to other persons, in their home, place of work, school or at play or in other aspects of their daily activities. The chapter does not encompass the attitudes of the person or people that are providing the support. The environmental factor being described is not the person or animal, but the amount of physical and emotional support the person or animal provides.

Chapter 4: Attitudes: This chapter is about the attitudes that are the observable consequences of customs, practices, ideologies, values, norms, factual beliefs and religious beliefs. These attitudes influence individual behaviour and social life at all levels, from interpersonal relationships and community associations to political, economic and legal structures; for example, individual or societal attitudes about a person’s trustworthiness and value as a human being that may motivate positive, honorific practices or negative and discriminatory practices (e.g. stigmatizing, stereotyping and marginalizing or neglect of the person). The attitudes classified are those of people external to the person whose situation is being described. They are not those of the person themselves. The individual attitudes are categorized according to the kinds of relationships listed in Environmental Factors Chapter 3. Values and beliefs are not coded separately from the attitudes as they are assumed to be the driving forces behind the attitudes.

Chapter 5: Services, systems and policies: This chapter is about:

1. Services that provide benefits, structured programmes and operations, in various sectors of society, designed to meet the needs of individuals. (Included in services are the people who provide them.) Services may be public, private or voluntary, and may be established at a local, community, regional, state, provincial, national or international level by individuals, associations, organizations, agencies or governments. The goods provided by these services may be general or adapted and specially designed.
2. Systems that are administrative control and organizational mechanisms, and are established by governments at the local, regional, national, and international levels, or by other recognized authorities. These systems are designed to organize, control and monitor services that provide benefits, structured programmes and operations in various sectors of society.
3. Policies constituted by rules, regulations, conventions and standards established by governments at the local, regional, national, and international levels, or by other recognized authorities. Policies govern and regulate the systems that organize, control and monitor services, structured programmes and operations in various sectors of society.

## Appendix 2

Attributes of the neighborhood environment not covered by previous selected studies on mobility or social participation in older adults

Chapter 1: Product and technology*

- e110: Products or substances for personal consumption
- e115:Products and technology for personal use in daily living
- e130: Products and technology for education
- e135: Products and technology for employment
- e145:Products and technology for the practice of religion and spirituality

Chapter 2:Natural environment and human-made changes to environment

- e230: Natural events
- e235: Human-caused events
- e255: Vibration

Chapter 3: Support and relationships

- e315: Extended family
- e330: People in positions of authority
- e335: People in subordinate positions
- e340: Personal care providers and personal assistants
- e355: Health professionals
- e360: Other professionals

Chapter 4: Attitudes

- e410: Individual attitudes of immediate family members
- e415: Individual attitudes of extended family members
- e420: Individual attitudes of friends
- e425:Individual attitudes of acquaintances, peers, colleagues, neighbors and community members
- e430:Individual attitudes of people in positions of authority
- e435:Individual attitudes of people in subordinate positions
- e440:Individual attitudes of personal care providers and personal assistants
- e450: Individual attitudes of health professionals
- e455: Individual attitudes of health-related professionals

Chapter 5: Services, systems and policies

- e510:Services, systems and policies for the production of consumer goods
- e520: Open space planning services, systems and policies
- e530: Utilities services, systems and policies
- e535: Communication services, systems and policies
- e550: Legal services, systems and policies
- e565: Economic services, systems and policies
- e570: Social security services, systems and policies
- e575:General social support services, systems and policies
- e585:Education and training services, systems and policies
- e590:Labour and employment services, systems and policies
- e595: Political services, systems and policies

*Based on the International Classification of Functioning, Disability and Health (ICF) [53]
